# Optimizing amino acid substitution matrices with a local alignment kernel

**DOI:** 10.1186/1471-2105-7-246

**Published:** 2006-05-05

**Authors:** Hiroto Saigo, Jean-Philippe Vert, Tatsuya Akutsu

**Affiliations:** 1 Bioinformatics Center, Institute for Chemical Research, Kyoto University, Uji, 611-0011, Japan; 2 Center for Computational Biology, Ecole des Mines de Paris, 35 rue Saint-Honoré, 77300 Fontainebleau, France

## Abstract

**Background:**

Detecting remote homologies by direct comparison of protein sequences remains a challenging task. We had previously developed a similarity score between sequences, called a *local alignment kernel*, that exhibits good performance for this task in combination with a support vector machine. The local alignment kernel depends on an amino acid substitution matrix. Since commonly used BLOSUM or PAM matrices for scoring amino acid matches have been optimized to be used in combination with the Smith-Waterman algorithm, the matrices optimal for the local alignment kernel can be different.

**Results:**

Contrary to the local alignment score computed by the Smith-Waterman algorithm, the local alignment kernel is differentiable with respect to the amino acid substitution and its derivative can be computed efficiently by dynamic programming. We optimized the substitution matrix by classical gradient descent by setting an objective function that measures how well the local alignment kernel discriminates homologs from non-homologs in the COG database. The local alignment kernel exhibits better performance when it uses the matrices and gap parameters optimized by this procedure than when it uses the matrices optimized for the Smith-Waterman algorithm. Furthermore, the matrices and gap parameters optimized for the local alignment kernel can also be used successfully by the Smith-Waterman algorithm.

**Conclusion:**

This optimization procedure leads to useful substitution matrices, both for the local alignment kernel and the Smith-Waterman algorithm. The best performance for homology detection is obtained by the local alignment kernel.

## Background

Sequence comparison for homology detection remains one of the core tools in bioinformatics. For example, BLAST [1] and PSI-BLAST [2] are widely used for this task, from wet biologists to bioinformaticians. Thanks to those tools, more than half of the newly identified protein sequences are nowadays recognized as having homologs [3]. The identification of *remote homologs*, however, remains a challenging task because sequence divergence can prevent sequence comparison algorithms from recognizing those homologies. In order to improve the performance of sequence comparison algorithms, a possible strategy is to use data from large databases like SCOP [4], PFAM [5] and COG [6] in order to optimize the parameters of the algorithm to detect homology. Following this strategy, we previously developed a score to compare protein sequences, called the *local alignment kernel *(LA kernel) [7], which in combination with a support vector machine could detect remote homology better than several state-of-the-art methods, including the Smith-Waterman (SW) algorithm [8], in a benchmark experiment based on the SCOP database. Although the LA kernel was used as a kernel function in combination with a support vector machine in [7], it can also be independently thought of as a measure of similarity between biological sequences, based on the scoring of local alignments between the sequences. In fact it bears similarities to the AAS algorithm [9], Hybrid Alignment algorithm [10] and BALSA algorithm [11] for sequence comparison, in the sense that all of these algorithms compute a summation of the scores over all possible local alignments (using a forward algorithm), instead of computing the score of only the best alignment (using the Viterbi algorithm), as the SW algorithm does.

Both the SW algorithm and the LA kernel depend critically on gap parameters and on a substitution matrix (also called a score matrix) that quantifies the contribution in the score of an alignment between any two given amino acids. Different substitution matrices lead to different alignment scores, and potentially to, different performance in terms of homology detection. Although homology detection is the actual goal of sequence comparison, most substitution matrices used in bioinformatics have been optimized for different purposes (a cluster of such matrices is available from the AAindex database [13]). For example, the PAM (point accepted mutation) matrices [14] are based on the probability of single point mutations and the theory of Markov chains. Among the PAM series the PAM250 matrix, which corresponds to the 250 PAM evolution time, is most frequently used in bioinformatics. Subsequently, Gonnet et al. [15] and Jones et al. [16] applied the same method to different and larger databases, resulting in different amino acid substitution matrices (GCB and JTT, respectively). The BLOSUM matrices [17] are constructed from the Blocks database of aligned protein sequences. The popular BLOSUM62 matrix is constructed from the blocks of sequence segments with identity larger than 62%.

A different methodology to construct a substitution matrix has been followed by Hourai et al. [18] and Kann et al. [19]. Following the idea that the final goal of sequence comparison is to detect homologies, these authors investigated the possibility to automatically optimize a substitution matrix to improve the performance of the final score in terms of homology detection. This optimization, based on a training dataset of pairs of proteins extracted from the Cluster of Orthologous Group (COG) database [6], uses an objective function that quantifies how well the final score separates the true homologs from non-homologs. Homology detection is known to be particularly difficult for pairs of proteins with less than 25% sequence identity, and the main motivations for these studies are to go further in this so-called "twilight zone" by assessing the performance of homology detection as the main objective function for the optimization of the substitution matrix. The methods of Hourai et al. and Kann et al. differ in the objective function that is optimized. Hourai et.al. try to separate the distribution of homologs from the distribution of non-homologs by minimizing the Bayes error rate. Kann et al. prepared a dataset of homologous pairs from the COG database and maximized the average *C*(confidence)-value of the pairs, where the *C *value is designed to be large when the expected number of non-homologous sequences scoring higher than the candidate pair is small. In spite of these differences, the methods by Hourai et al. and Kann et al. both suffer from the difficulty to optimize the SW score with respect to the substitution matrix. Indeed, the fact that the SW score only takes into account the maximum scoring alignment makes it non-differentiable with respect to the substitution matrix. As a result, the final objective function which is based on SW scores is itself not differentiable with respect to the substitution matrix, and therefore difficult to optimize. The trick used by both algorithms is to observe that the SW score of a pair of sequences is piecewise differentiable, as long as the maximum scoring alignment remains the same. Hence the authors suggest to alternate both local optimization of the substitution matrix by gradient descent and computation of the best scoring alignment that depends on the current substitution matrix. A drawback of this approach is that the local moves of the substitution matrices, based on a given set of alignments, might be very different from those required to globally optimize the objective function.

This paper is devoted to the extension of these approaches to the LA kernel, instead of the SW local alignment score. The motivations for this work are twofold. First, the LA kernel was previously shown to be a more sensitive measure of similarity for remote homologs, suggesting that it could also remain competitive with an optimized substitution matrix. Second, contrary to the SW score, the LA kernel is differentiable with respect to the elements of the substitution matrix and the gap parameters, and we show below that these derivatives can be computed efficiently by dynamic programming. This means that any objective function that is itself differentiable with respect to the LA kernel is differentiable with respect to the substitution matrix and can be optimized by simple gradient descent methods, without the need to alternate between the gradient descent steps and alignment steps used in the optimization of the SW score. Applying this procedure to the objective function used in [19], we optimized the substitution matrix as well as the gap parameters to separate true homologs from non-homologs in a dataset of protein sequences extracted from the COG database, and evaluated the performance of the resulting methods for homology detection on several independent test sets. We compared these results with those obtained after optimizing the substitution matrix with the Smith-Waterman algorithm [19], and compared how each scoring algorithm performs with each optimized matrix.

## Results

Pairs of homologous sequences with identity smaller than 20% were collected from the COG database and used for the training and testing of the method. For each pair, an *E-*value measuring the significance of the alignment score was computed, from which the corresponding confidence value *C = *1/(1 +* E*) was derived. The objective of the optimization procedure is to maximize the mean confidence value ⟨*C*⟩ over the training set, and its performance is evaluated by the average confidence value on the test set. In order to avoid the risk of falling into local optima, we used several amino acid substitution matrices (BLOSUM62, PAM250, JTT, GCB) with default gap parameters (12 and 2 for gap open and extension penalties, respectively) as starting points of the optimization. Among them, BLOSUM62 led to the best local optimum, and we present the performance of this optimization below.

### Improvement of confidence values for the SW algorithm and the LA kernel

**Figure 1:**
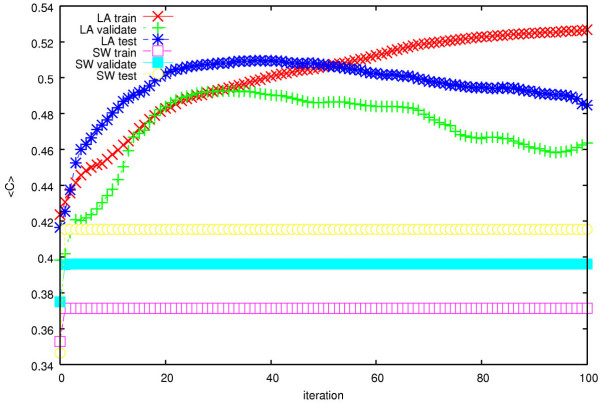
**Learning curve**. ⟨*C*⟩ values averaged over training, validation and testing data during the optimization process.

**Figure 2:**
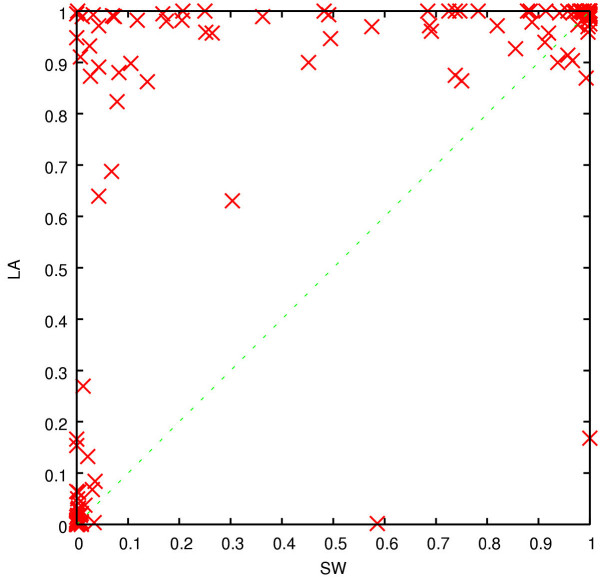
**Comparison of *C *values for the LA kernel and the the SW algorithm**. In the graph, LA indicates *C *values of the LA kernel with BLOSUM62LAOPT, and SW indicates *C *values of the SW algorithm with BLOSUM62SWOPT. If the two methods perform equally, every point would be aligned along the diagonal. More points distributed in the upper-left triangle shows the better performance of the LA kernel with BLOSUM62LAOPT.

The mean confidence values ⟨*C*⟩ over the 300 training, 48 validation and 47 test pairs during the optimization procedure for both the LA kernel and the SW score are plotted in Figure 1. The optimization was carried out on the training set until the criterion reached a maximum on the validation set, to prevent over-fitting of the parameters to the training set. The performance of this procedure was then evaluated on the independent test set. As expected, we observe that the confidence value on the training set smoothly increases during the optimization. In the case of the LA kernel, a maximum is reached around 30 iterations in the validation set. The mean *C *value on the test set also seems to have reached its maximum around 30 iterations. The learning curve for the SW score also increases on the training, validation and test sets, and reaches a maximum after the first iteration. However, we observed that the convergence is not always as fast when starting from different substitution matrices (data not shown). This figure also demonstrates that the optimization with the LA kernel goes further than with the SW algorithm in terms of mean *C *value. Figure 2 is the comparison of the optimized *C *values for the LA kernel and the SW algorithm. In the graph, each point corresponds to a training pair of sequeces. This graphs illustrate the fact that the optimization process progresses further for the LA kernel than for the SW algorithm, and that many pairs (although not all) reach a remarkable confidence value.

**Figure 3:**
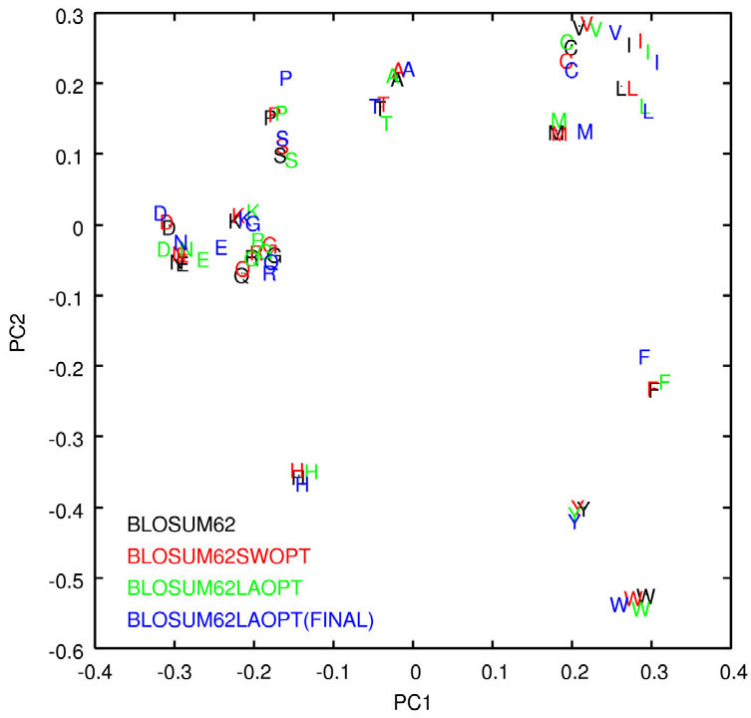
**Transition of score matrix through optimization**. Principal components projection along the two principal eigenvectors for the distribution of BLOSUM62, BLOSUM62SWOPT, BLOSUM62LAOPT and BLOSUM62LAOPT(FINAL). The overall placement of each amino acid residue was roughly similar through the optimized matrices.

The amino acid substitution matrices as well as gap parameters optimized for the SW score (BLOSUM62SWOPT) and the LA kernel (BLOSUM62LAOPT) on the training set are shown in Tables 1 and 2, respectively. It should be noted that the score matrix optimized for the SW score is based on Kann et al.'s method, and the score matrix optimized for the LA kernel is based on our method. In spite of a global conservation of most values, slight variations can be observed. To see the difference of those slight changes, we performed a principal component analysis (PCA) for each obtained matrix and plotted along the 1st and the 2nd principal components (Figure 3).

Moreover, we calculated the average *l*_1_-distance between two matrices *M*_1 _and *M*_2 _as

D(M1,M2)=1|a||b|∑a,b|M1(a,b)−M2(a,b)|,
 MathType@MTEF@5@5@+=feaafiart1ev1aaatCvAUfKttLearuWrP9MDH5MBPbIqV92AaeXatLxBI9gBaebbnrfifHhDYfgasaacH8akY=wiFfYdH8Gipec8Eeeu0xXdbba9frFj0=OqFfea0dXdd9vqai=hGuQ8kuc9pgc9s8qqaq=dirpe0xb9q8qiLsFr0=vr0=vr0dc8meaabaqaciaacaGaaeqabaqabeGadaaakeaacqWGebardaqadaqaaiabd2eannaaBaaaleaacqaIXaqmaeqaaOGaeiilaWIaemyta00aaSbaaSqaaiabikdaYaqabaaakiaawIcacaGLPaaacqGH9aqpdaWcaaqaaiabigdaXaqaamaaemaabaGaemyyaegacaGLhWUaayjcSdWaaqWaaeaacqWGIbGyaiaawEa7caGLiWoaaaWaaabuaeaadaabdaqaaiabd2eannaaBaaaleaacqaIXaqmaeqaaOWaaeWaaeaacqWGHbqycqGGSaalcqWGIbGyaiaawIcacaGLPaaacqGHsislcqWGnbqtdaWgaaWcbaGaeGOmaidabeaakmaabmaabaGaemyyaeMaeiilaWIaemOyaigacaGLOaGaayzkaaaacaGLhWUaayjcSdaaleaacqWGHbqycqGGSaalcqWGIbGyaeqaniabggHiLdGccqGGSaalaaa@58C6@

where *M*(*a*, *b *)denotes the substitution score between amino acids *a *and *b, |a| *and |*b*| are the number of amino acids (= 20). The averaged *l*_1_-distances between BLOSUM62 and BLOSUM62SWOPT, BLOSUM62 and BLOSUM62LAOPT, and finally BLOSUM62SWOPT and BLOSUM62LAOPT, are 0.074, 0.26 and 0.23, respectively. These differences show that the optimization with the LA kernel diverged further from the original matrix (0.26) than with the SW score (0.23), and that both optimizations did not necessarily go in the same direction. The matrix shown in Table 3 is the final matrix optimized for the LA kernel using both the COG training and test data. The *l*_1_-distance between this matrix and the original BLOSUM62 matrix is 0.30, and the result of PCA on this matrix is shown in Figure 3. The overall placement of each amino acid residue was similar through the matrices, reflecting physicochemical properties of the different amino-acids. For example, charged (D, E, K, R, H) or polar amino acids (S, T, P, N, Q) are placed on the left side of the figure, while non-polar amino acids (A, C, L, M, I, V, F, W) are placed on the right side. One exception is glycine (G), which is known to be a non-polar amino acid but is placed within the cluster of polar amino acids. This may be because of its conformational flexibility with only a proton constituting its side chain. For PC2, we can observe that amino acids with rings (H, Y, F, W) are placed distinctly from other amino acids.

The reason why the optimized matrix at first sight look very similar to the BLOSUM62 matrix is certainly that the latter is already a very good substitution matrix extensively used by the research community for homology detection. The slight differences in the substitution matrices, however, lead to significant improvements in the mean ⟨*C*⟩ value.

### Results on independent test sets

**Figure 4:**
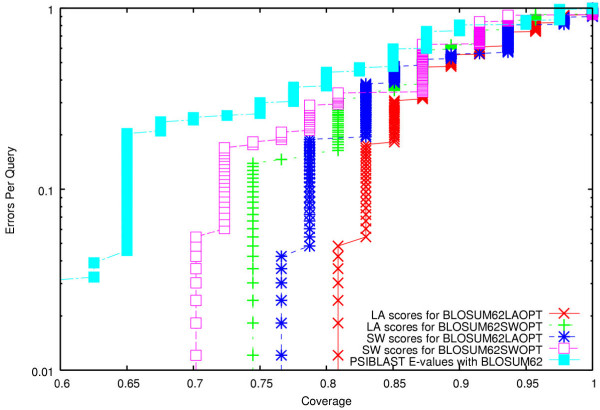
**Coverage vs Error Per Query plots for the COG distant (identity less than 20%) test set**. In the graph, BLOSUM62SWOPT and BLOSUM62LAOPT mean the amino acid substitution matrices optimized starting from BLOSUM62 with the SW algorithm and the LA kernel, respectively. SW scores and LA scores mean the *E*-values of the SW algorithm and the LA kernel algorithm, respectively. All of the proteins in the COG test set were compared with each other using the SW algorithm or the LA kernel with BLOSUM62LAOPT or BLOSUM62SWOPT. Curves located further to the right side indicate better performance.

Performances of algorithms over the COG test set were evaluated for both the LA kernel and the SW score in combination with both BLOSUM62LAOPT and BLOSUM62SWOPT. Figure 4 shows the Errors Per Query plot proposed by Brenner et al. [20], where coverage was defined as the fraction of true homologs that have scores above the threshold, and errors per query was defined as the number of non-homologous pairs above the threshold divided by the number of queries. This approach is closely related to ROC (Receiver Operating Characterisitc) analysis: coverage can be considered as a fraction of true positives and errors per query is a rate of false positives. More "Coverage" with less "Errors Per Query" means better performance. Therefore the methods with the left-most distributions in this plot can be thought of as being capable of going deeper into the twilight zone, in terms of of detecting remote homologs. The normalized area under the ROC curve is also used as a common measure to compare the performance of *ranking*, and takes values between 0 and 1. An ROC score of 1 means a perfect ranking, and an ROC score close to 0.5 means almost as good as random. The ROC score for each method is shown in Table 4. In the table, the result of PSI-BLAST [2] with the BLOSUM62 matrix was shown together as a baseline method. PSI-BLAST was trained using the training dataset only with the option "-j 2". In order to evaluate the performance of the score matrix, no large external database was used to make a profile for PSI-BLAST, since incorporating a huge database into a profile eliminates the effect of a initial score matrix. The ROC score for PSI-BLAST with BLOSUM62 was 0.811 in the COG distant test set. For the LA kernel, they were 0.852 and 0.895 with BLOSUM62 and BLOSUM62OPT, respectively. The ROC score for PSI-BLAST with BLOSUM62 was 0.854 in the PFAM distant test set. For LA kernel, they were 0.932 and 0.947 with BLOSUM62 and BLOSUM62OPT, respectively. This shows that the gain in performance resulting from the use of the LA kernel instead of the PSI-BLAST method is in the range 5.1% – 9.1%, while the gain resulting from the optimization of the substitution matrix with the LA kernel is in the range 1.6% – 5.1%. Overall the performance improvement resulting from the use of the LA kernel with BLOSUM62LAOPT over the standard PSI-BLAST method with BLOSUM62 is around 10% for the distant test sets, and almost zero for the close test sets in terms of ROC score (Table 4).

**Figure 5:**
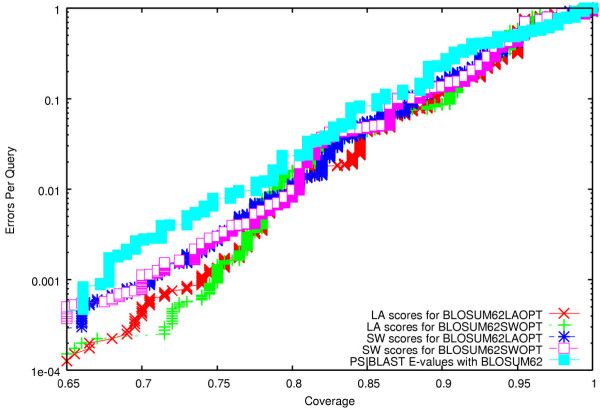
**Coverage vs Error Per Query plots for the PFAM distant (identity less than 20%) test set**. In the graph, BLOSUM62SWOPT and BLOSUM62LAOPT mean the amino acid substitution matrices optimized starting from BLOSUM62 with the SW algorithm and the LA kernel, respectively. SW scores and LA scores mean the *E*-values of the SW algorithm and the LA kernel algorithm, respectively. All of the proteins in the PFAM distant test set were compared with each other using the SW algorithm or the LA kernel with BLOSUM62LAOPT or BLOSUM62SWOPT. Curves located further to the right side indicate better performance.

With these criteria, the LA kernel based on the BLOSUM62LAOPT substitution matrix is the most effective. Interestingly, the BLOSUM62LAOPT matrix is as good as the BLOSUM62SWOPT matrix when used with the SW algorithm as well, although the BLOSUM62LAOPT matrix was optimized with the LA kernel. This highlights the fact that optimization based on the SW score encounters more difficulty in finding a good maximum than the optimization based on the LA kernel. Finally we observe that the LA kernel outperforms the SW algorithm also in the independent PFAM distant test set (Figure 5) with both optimized matrices, confirming its superiority over a large choice of parameters.

**Figure 6:**
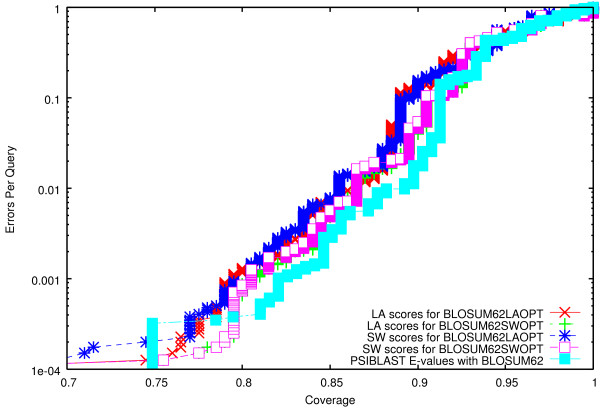
**Coverage vs Error Per Query plots for the COG close (identity 25–40%) test set**. In the graph, BLOSUM62SWOPT and BLOSUM62LAOPT mean the amino acid substitution matrices optimized starting from BLOSUM62 with the SW algorithm and the LA kernel, respectively. SW scores and LA scores mean the *E*-values of the SW algorithm and the LA kernel algorithm, respectively. All of the proteins in the COG close test set were compared with each other using the SW algorithm or the LA kernel with BLOSUM62LAOPT or BLOSUM62SWOPT. Curves located further to the right side indicate better performance.

**Figure 7:**
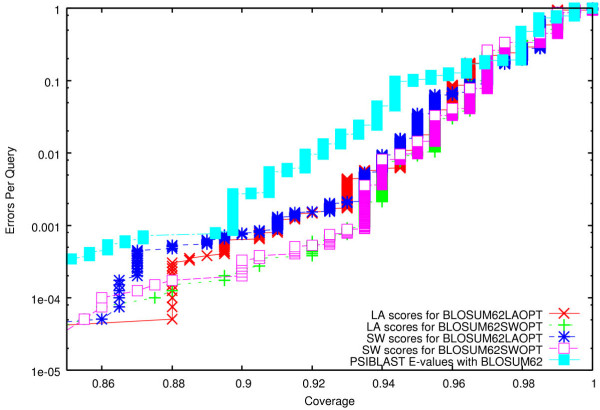
**Coverage vs Error Per Query plots for the PFAM close (identity 25–40%) test set**. In the graph, BLOSUM62SWOPT and BLOSUM62LAOPT mean the amino acid substitution matrices optimized starting from BLOSUM62 with the SW algorithm and the LA kernel, respectively. SW scores and LA scores mean the *E*-values of the SW algorithm and the LA kernel algorithm, respectively. All of the proteins in the PFAM close test set were compared with each other using the SW algorithm or the LA kernel with BLOSUM62LAOPT or BLOSUM62SWOPT. Curves located further to the right side indicate better performance.

Interestingly, although the BLOSUM62LAOPT matrix and BLOSUM62SWOPT matrix are optimized for the detection of remote homologs, they also performed competitively in the COG and PFAM close homologs dataset (Figure 6, 7). The average *C *value that is calculated for the whole test dataset with various optimized matrices in combination with the SW algorithm and the LA kernel is shown in Table 5. Results of an ROC analysis for each method in each database are also shown in Table 4. We can observe that in the dataset of close homologs, the differences of performance are much smaller than those in the dataset of distant homologs. Interestingly, the superiority of this method is particularly important in the case of the PFAM distant test set (Figure 5), that is, in the detection of remote homologs. This can certainly be attributed to the fact that the training set itself (COG distant) was made of distant homologs, and suggests that different substitution matrices might be optimized for different levels of sequence similarities.

## Discussion

The main contribution of this paper is to propose an optimization framework for substitution matrices based on an exact gradient descent method. This approach is made possible by the fact that the alignment score we consider, the LA kernel, is differentiable with respect to the substitution matrix, contrary to the SW alignment score. The fact that the matrix optimized with this approach outperforms the matrix optimized with the SW score even for the SW algorithm itself suggests that there is an important benefit for the LA kernel approach compared to the more heuristic nature of the optimization in the case of the SW score. It should be pointed out, however, that there is a continuum between the LA kernel and the SW score [7]. Indeed the LA kernel depends on a parameter *β *set to 0.5 in this study, but increasing *β *high enough finally leads to the SW score. In other words, the SW score, which is piecewise linear with respect to the elements of the substitution matrix (and therefore only piecewise differentiable), can be seen as the limit of infinitely differentiable functions. Intuitively, the optimization of the LA kernel can be thought of as the optimization of a smooth approximation of the SW score, which can more easily find good local optima. This suggests, in the spirit of simulated annealing, that further improvements for the SW algorithm might be obtained by optimizing the LA kernel and increasing *β *simultaneously; however, we leave this avenue of research for future work.

It should be pointed out that fixing the scaling parameter *β *to 0.5 is not a restriction in itself. Indeed, although the derivative of the LA kernel with respect to *β *can also be computed efficiently by dynamic programming, leading to the possibility of optimizing *β *as well as the substitution matrix and gap parameters, this would have no effect on the optimal score function that only depends on the products of *β *with the substitution and gap parameters. In other words, allowing *β *to vary would lead to an over-parameterized model. Although fixing *β *has therefore no effect on the global optimum of the objective function, it might nevertheless have an important effect on our optimization procedure because it defines where the optimization starts. Taking *β *= 0.5 with default gap and substitution parameter, which were shown in previous studies to perform well on remote homology detection, is certainly a safe choice as starting point of the optimization.

A second point to be highlighted is the good performance of the LA kernel as a similarity score compared to the SW score. While it was shown in [7] that the LA kernel outperforms the SW score as a kernel function for support vector machines, the present studies validate the relevance of the LA kernel as a measure of similarity. It should be pointed out that the advantage of the LA kernel over the SW score is expected to increase for remote homologs, because when the sequence identity is small the best local alignment computed by the SW score is likely not to be the correct one, and in this case multiple hits of relatively short suboptimal alignments (*motifs *)between two sequences would be of importance, leading to the idea that averaging scores over a large number of candidate alignments might provide better evidence for homology [11].

Finally, let us mention that the LA kernel is in fact infinitely differentiable, and its second derivative (Hessian) with respect to the substitution matrix could be computed, also by dynamic programming. It would therefore be possible, in principle, to use faster gradient descent algorithms such as Newton's method for the optimization. We did not follow this avenue in our experiments because this would require the computation of a 212 × 212 Hessian matrix at each iteration, which would need more than 100 times the amount of computation than without the computation of the Hessian. Of course, the Hessian is of no help for the SW algorithm, because it is constantly equal to zero on the points where the SW score is differentiable.

## Conclusion

We proposed a method to optimize amino acid substitution matrices for the LA kernel, based on the properties of differentiability of the LA kernel with respect to the substitution matrix. This is the first time amino acid substitution matrices for pairwise sequence comparison are optimized for use with the forward algorithm [12]. The optimized matrices exhibit good performance on distant datasets both with the SW algorithm and the LA kernel, and they are competitive on close datasets. The derived matrices may be useful when standard methods fail to detect homologs.

## Methods

In this section, we first show how to compute the derivative of the LA kernel with respect to the elements of an amino acid substitution matrix. Then we present an objective function meant to favor the discrimination between true homologs and non-homologs, and finally explain how we created the datasets used in this study.

### The LA kernel

The LA kernel [7] between two sequences **x **and **y **is defined by

KLA(x,y)=∑πeβs(x,y,π),     (1)
 MathType@MTEF@5@5@+=feaafiart1ev1aaatCvAUfKttLearuWrP9MDH5MBPbIqV92AaeXatLxBI9gBaebbnrfifHhDYfgasaacH8akY=wiFfYdH8Gipec8Eeeu0xXdbba9frFj0=OqFfea0dXdd9vqai=hGuQ8kuc9pgc9s8qqaq=dirpe0xb9q8qiLsFr0=vr0=vr0dc8meaabaqaciaacaGaaeqabaqabeGadaaakeaacqWGlbWsdaWgaaWcbaGaemitaWKaemyqaeeabeaakmaabmaabaacbeGae8hEaGNaeiilaWIae8xEaKhacaGLOaGaayzkaaGaeyypa0ZaaabuaeaacqWGLbqzdaahaaWcbeqaaGGaciab+j7aIjabdohaZnaabmaabaGae8hEaGNaeiilaWIae8xEaKNaeiilaWIae4hWdahacaGLOaGaayzkaaaaaaqaaiab+b8aWbqab0GaeyyeIuoakiabcYcaSiaaxMaacaWLjaWaaeWaaeaacqaIXaqmaiaawIcacaGLPaaaaaa@4B8A@

where *β *is a parameter, *π *runs over the possible local alignments between **x **and **y**, and *s*(**x**, **y**, *π*) is the score of an alignment *π *between *x *and *y*. The score of an alignment *π *is itself given by the sum of substitution scores for the letters paired together, minus an affine gap penalty:

s(x,y,π)=∑a,bna,b(x,y,π)S(a,b)−ngd(x,y,π)gd−nge(x,y,π)ge,
 MathType@MTEF@5@5@+=feaafiart1ev1aaatCvAUfKttLearuWrP9MDH5MBPbIqV92AaeXatLxBI9gBaebbnrfifHhDYfgasaacH8akY=wiFfYdH8Gipec8Eeeu0xXdbba9frFj0=OqFfea0dXdd9vqai=hGuQ8kuc9pgc9s8qqaq=dirpe0xb9q8qiLsFr0=vr0=vr0dc8meaabaqaciaacaGaaeqabaqabeGadaaakeaacqWGZbWCdaqadaqaaGqabiab=Hha4jabcYcaSiab=Lha5jabcYcaSGGaciab+b8aWbGaayjkaiaawMcaaiabg2da9maaqafabaGaemOBa42aaSbaaSqaaiabdggaHjabcYcaSiabdkgaIbqabaGcdaqadaqaaiab=Hha4jabcYcaSiab=Lha5jabcYcaSiab+b8aWbGaayjkaiaawMcaaiabdofatnaabmaabaGaemyyaeMaeiilaWIaemOyaigacaGLOaGaayzkaaGaeyOeI0IaemOBa42aaSbaaSqaaiabdEgaNnaaBaaameaacqWGKbazaeqaaaWcbeaakmaabmaabaGae8hEaGNaeiilaWIae8xEaKNaeiilaWIae4hWdahacaGLOaGaayzkaaGaem4zaC2aaSbaaSqaaiabdsgaKbqabaGccqGHsislcqWGUbGBdaWgaaWcbaGaem4zaC2aaSbaaWqaaiabdwgaLbqabaaaleqaaOWaaeWaaeaacqWF4baEcqGGSaalcqWF5bqEcqGGSaalcqGFapaCaiaawIcacaGLPaaacqWGNbWzdaWgaaWcbaGaemyzaugabeaaaeaacqWGHbqycqGGSaalcqWGIbGyaeqaniabggHiLdGccqGGSaalaaa@712A@

where *n*_*a*,*b*_(**x**, **y**, *π*) represents the number of times that the amino acid *a *is aligned with the amino acid *b*, *S*(*a*, *b*) denotes the substitution score between amino acids *a *and *b*, *n*_*gd*_(**x**, **y**,*π*) and *n*_*ge*_(**x**, **y**, *π*) are the number of gap opens and extensions, respectively, and *g*_*d *_and *g*_*e *_are penalties for gap open and gap extension, respectively.

As shown in (1) the LA kernel takes into account all possible alignments between two strings by summing the scores, and can be computed by the following algorithm.

### Algorithm 1: local alignment kernel

Initialization:for  i=0,…,|x|  and  j=0,…,|y|:Mi,0=M0,j=Xi,0=X0,j=X2i,0=X20,j=Yi,0=Y0,j=Y2i,0=Y20,j=0,Iteration:for  i=1,…,|x|  and  j=1,…,|y|:Mi,j=eβS(xi,yi)(1+Xi−1,j−1+Yi−1,j−1+Mi−1,j−1),Xi,j=eβgd(Mi−1,j)+eβge(Xi−1,j),Yi,j=eβgd(Mi,j−1+Xi,j−1)+eβge(Yi,j−1),X2i,j=Mi−1,j+X2i−1,j,Y2i,j=Mi,j−1+X2i,j−1+Y2i,j−1,Termination:KLA(x,y)=1+X2|x|,|y|+Y2|x|,|y|+M|x|,|y|.
 MathType@MTEF@5@5@+=feaafiart1ev1aaatCvAUfKttLearuWrP9MDH5MBPbIqV92AaeXatLxBI9gBaebbnrfifHhDYfgasaacH8akY=wiFfYdH8Gipec8Eeeu0xXdbba9frFj0=OqFfea0dXdd9vqai=hGuQ8kuc9pgc9s8qqaq=dirpe0xb9q8qiLsFr0=vr0=vr0dc8meaabaqaciaacaGaaeqabaqabeGadaaakeaafaqaaeGcdaaaaaqaaiabbMeajjabb6gaUjabbMgaPjabbsha0jabbMgaPjabbggaHjabbYgaSjabbMgaPjabbQha6jabbggaHjabbsha0jabbMgaPjabb+gaVjabb6gaUbqaaiabbQda6aqaaiabbAgaMjabb+gaVjabbkhaYjabbccaGiabbccaGiabdMgaPjabg2da9iabicdaWiabcYcaSiablAciljabcYcaSmaaemaabaacbeGae8hEaGhacaGLhWUaayjcSdGaeeiiaaIaeeiiaaIaeeyyaeMaeeOBa4MaeeizaqMaeeiiaaIaeeiiaaIaemOAaOMaeyypa0JaeGimaaJaeiilaWIaeSOjGSKaeiilaWYaaqWaaeaacqWF5bqEaiaawEa7caGLiWoacqGG6aGoaeGabaaGeiaaxMaacqWGnbqtdaWgaaWcbaGaemyAaKMaeiilaWIaeGimaadabeaaaOqaaiabg2da9aqaaiabd2eannaaBaaaleaacqaIWaamcqGGSaalcqWGQbGAaeqaaOGaeyypa0JaemiwaG1aaSbaaSqaaiabdMgaPjabcYcaSiabicdaWaqabaGccqGH9aqpcqWGybawdaWgaaWcbaGaeGimaaJaeiilaWIaemOAaOgabeaakiabg2da9iabdIfayjabikdaYmaaBaaaleaacqWGPbqAcqGGSaalcqaIWaamaeqaaOGaeyypa0JaemiwaGLaeGOmaiZaaSbaaSqaaiabicdaWiabcYcaSiabdQgaQbqabaGccqGH9aqpcqWGzbqwdaWgaaWcbaGaemyAaKMaeiilaWIaeGimaadabeaakiabg2da9iabdMfaznaaBaaaleaacqaIWaamcqGGSaalcqWGQbGAaeqaaOGaeyypa0JaemywaKLaeGOmaiZaaSbaaSqaaiabdMgaPjabcYcaSiabicdaWaqabaGccqGH9aqpcqWGzbqwcqaIYaGmdaWgaaWcbaGaeGimaaJaeiilaWIaemOAaOgabeaakiabg2da9iabicdaWiabcYcaSaqaceaayjGaaCzcaiabbMeajjabbsha0jabbwgaLjabbkhaYjabbggaHjabbsha0jabbMgaPjabb+gaVjabb6gaUbqaaiabbQda6aqaaiabbAgaMjabb+gaVjabbkhaYjabbccaGiabbccaGiabdMgaPjabg2da9iabigdaXiabcYcaSiablAciljabcYcaSmaaemaabaGae8hEaGhacaGLhWUaayjcSdGaeeiiaaIaeeiiaaIaeeyyaeMaeeOBa4MaeeizaqMaeeiiaaIaeeiiaaIaemOAaOMaeyypa0JaeGymaeJaeiilaWIaeSOjGSKaeiilaWYaaqWaaeaacqWF5bqEaiaawEa7caGLiWoacqGG6aGoaeGabaaMeiaaxMaacqWGnbqtdaWgaaWcbaGaemyAaKMaeiilaWIaemOAaOgabeaaaOqaaiabg2da9aqaaiabdwgaLnaaCaaaleqabaacciGae4NSdiMaem4uam1aaeWaaeaacqWG4baEdaWgaaadbaGaemyAaKgabeaaliabcYcaSiabdMha5naaBaaameaacqWGPbqAaeqaaaWccaGLOaGaayzkaaaaaOWaaeWaaeaacqaIXaqmcqGHRaWkcqWGybawdaWgaaWcbaGaemyAaKMaeyOeI0IaeGymaeJaeiilaWIaemOAaOMaeyOeI0IaeGymaedabeaakiabgUcaRiabdMfaznaaBaaaleaacqWGPbqAcqGHsislcqaIXaqmcqGGSaalcqWGQbGAcqGHsislcqaIXaqmaeqaaOGaey4kaSIaemyta00aaSbaaSqaaiabdMgaPjabgkHiTiabigdaXiabcYcaSiabdQgaQjabgkHiTiabigdaXaqabaaakiaawIcacaGLPaaacqGGSaalaeGabaaPeiaaxMaacqWGybawdaWgaaWcbaGaemyAaKMaeiilaWIaemOAaOgabeaaaOqaaiabg2da9aqaaiabdwgaLnaaCaaaleqabaGae4NSdiMaem4zaC2aaSbaaWqaaiabdsgaKbqabaaaaOWaaeWaaeaacqWGnbqtdaWgaaWcbaGaemyAaKMaeyOeI0IaeGymaeJaeiilaWIaemOAaOgabeaaaOGaayjkaiaawMcaaiabgUcaRiabdwgaLnaaCaaaleqabaGae4NSdiMaem4zaC2aaSbaaWqaaiabdwgaLbqabaaaaOWaaeWaaeaacqWGybawdaWgaaWcbaGaemyAaKMaeyOeI0IaeGymaeJaeiilaWIaemOAaOgabeaaaOGaayjkaiaawMcaaiabcYcaSaqaceaa8sGaaCzcaiabdMfaznaaBaaaleaacqWGPbqAcqGGSaalcqWGQbGAaeqaaaGcbaGaeyypa0dabaGaemyzau2aaWbaaSqabeaacqGFYoGycqWGNbWzdaWgaaadbaGaemizaqgabeaaaaGcdaqadaqaaiabd2eannaaBaaaleaacqWGPbqAcqGGSaalcqWGQbGAcqGHsislcqaIXaqmaeqaaOGaey4kaSIaemiwaG1aaSbaaSqaaiabdMgaPjabcYcaSiabdQgaQjabgkHiTiabigdaXaqabaaakiaawIcacaGLPaaacqGHRaWkcqWGLbqzdaahaaWcbeqaaiab+j7aIjabdEgaNnaaBaaameaacqWGLbqzaeqaaaaakmaabmaabaGaemywaK1aaSbaaSqaaiabdMgaPjabcYcaSiabdQgaQjabgkHiTiabigdaXaqabaaakiaawIcacaGLPaaacqGGSaalaeGabaaWdiaaxMaacqWGybawcqaIYaGmdaWgaaWcbaGaemyAaKMaeiilaWIaemOAaOgabeaaaOqaaiabg2da9aqaaiabd2eannaaBaaaleaacqWGPbqAcqGHsislcqaIXaqmcqGGSaalcqWGQbGAaeqaaOGaey4kaSIaemiwaGLaeGOmaiZaaSbaaSqaaiabdMgaPjabgkHiTiabigdaXiabcYcaSiabdQgaQbqabaGccqGGSaalaeGabaa2diaaxMaacqWGzbqwcqaIYaGmdaWgaaWcbaGaemyAaKMaeiilaWIaemOAaOgabeaaaOqaaiabg2da9aqaaiabd2eannaaBaaaleaacqWGPbqAcqGGSaalcqWGQbGAcqGHsislcqaIXaqmaeqaaOGaey4kaSIaemiwaGLaeGOmaiZaaSbaaSqaaiabdMgaPjabcYcaSiabdQgaQjabgkHiTiabigdaXaqabaGccqGHRaWkcqWGzbqwcqaIYaGmdaWgaaWcbaGaemyAaKMaeiilaWIaemOAaOMaeyOeI0IaeGymaedabeaakiabcYcaSaqaaiabbsfaujabbwgaLjabbkhaYjabb2gaTjabbMgaPjabb6gaUjabbggaHjabbsha0jabbMgaPjabb+gaVjabb6gaUbqaaiabbQda6aqaaaqaceaaOfGaaCzcaiabdUealnaaBaaaleaacqWGmbatcqWGbbqqaeqaaOWaaeWaaeaacqWF4baEcqWFSaalcqWF5bqEaiaawIcacaGLPaaaaeaacqGH9aqpaeaacqaIXaqmcqGHRaWkcqWGybawcqaIYaGmdaWgaaWcbaWaaqWaaeaacqWF4baEaiaawEa7caGLiWoacqGGSaaldaabdaqaaiab=Lha5bGaay5bSlaawIa7aaqabaGccqGHRaWkcqWGzbqwcqaIYaGmdaWgaaWcbaWaaqWaaeaacqWF4baEaiaawEa7caGLiWoacqGGSaaldaabdaqaaiab=Lha5bGaay5bSlaawIa7aaqabaGccqGHRaWkcqWGnbqtdaWgaaWcbaWaaqWaaeaacqWF4baEaiaawEa7caGLiWoacqGGSaaldaabdaqaaiab=Lha5bGaay5bSlaawIa7aaqabaGccqGGUaGlaaaaaa@DA40@

In the above algorithm, *M *stands for the matching state between amino acids, while *X, Y*, *X*2 and *Y2 *are for the states corresponding to insertions or deletions.

The score of the LA kernel is then described as the logarithm of (1):

K^LA(x,y)=log⁡KLA(x,y)=log⁡∑πeβs(x,y,π).     (2)
 MathType@MTEF@5@5@+=feaafiart1ev1aaatCvAUfKttLearuWrP9MDH5MBPbIqV92AaeXatLxBI9gBaebbnrfifHhDYfgasaacH8akY=wiFfYdH8Gipec8Eeeu0xXdbba9frFj0=OqFfea0dXdd9vqai=hGuQ8kuc9pgc9s8qqaq=dirpe0xb9q8qiLsFr0=vr0=vr0dc8meaabaqaciaacaGaaeqabaqabeGadaaakeaacuWGlbWsgaqcamaaBaaaleaacqWGmbatcqWGbbqqaeqaaOWaaeWaaeaaieqacqWF4baEcqWFSaalcqWF5bqEaiaawIcacaGLPaaacqGH9aqpcyGGSbaBcqGGVbWBcqGGNbWzcqWGlbWsdaWgaaWcbaGaemitaWKaemyqaeeabeaakmaabmaabaGae8hEaGNae8hlaWIae8xEaKhacaGLOaGaayzkaaGaeyypa0JagiiBaWMaei4Ba8Maei4zaC2aaabuaeaacqWGLbqzdaahaaWcbeqaaGGaciab+j7aIjabdohaZnaabmaabaGae8hEaGNae8hlaWIae8xEaKNae8hlaWIae4hWdahacaGLOaGaayzkaaaaaaqaaiab+b8aWbqab0GaeyyeIuoakiabc6caUiaaxMaacaWLjaWaaeWaaeaacqaIYaGmaiaawIcacaGLPaaaaaa@5DAC@

The derivative of (2) with respect to *S(a, b) *can therefore be written as:

∂K^LA(x,y)∂S(a,b)=∂∂S(a,b)∑πeβs(x,y,π)∑πeβs(x,y,π).     (3)
 MathType@MTEF@5@5@+=feaafiart1ev1aaatCvAUfKttLearuWrP9MDH5MBPbIqV92AaeXatLxBI9gBaebbnrfifHhDYfgasaacH8akY=wiFfYdH8Gipec8Eeeu0xXdbba9frFj0=OqFfea0dXdd9vqai=hGuQ8kuc9pgc9s8qqaq=dirpe0xb9q8qiLsFr0=vr0=vr0dc8meaabaqaciaacaGaaeqabaqabeGadaaakeaadaWcaaqaaiabgkGi2kqbdUealzaajaWaaSbaaSqaaiabdYeamjabdgeabbqabaGcdaqadaqaaGqabiab=Hha4jabcYcaSiab=Lha5bGaayjkaiaawMcaaaqaaiabgkGi2kabdofatnaabmaabaGaemyyaeMaeiilaWIaemOyaigacaGLOaGaayzkaaaaaiabg2da9maalaaabaWaaSaaaeaacqGHciITaeaacqGHciITcqWGtbWudaqadaqaaiabdggaHjabcYcaSiabdkgaIbGaayjkaiaawMcaaaaadaaeqaqaaiabdwgaLnaaCaaaleqabaacciGae4NSdiMaem4Cam3aaeWaaeaacqWF4baEcqGGSaalcqWF5bqEcqWFSaalcqGFapaCaiaawIcacaGLPaaaaaaabaGae4hWdahabeqdcqGHris5aaGcbaWaaabeaeaacqWGLbqzdaahaaWcbeqaaiab+j7aIjabdohaZnaabmaabaGae8hEaGNaeiilaWIae8xEaKNae8hlaWIae4hWdahacaGLOaGaayzkaaaaaaqaaiab+b8aWbqab0GaeyyeIuoaaaGccqGGUaGlcaWLjaGaaCzcamaabmaabaGaeG4mamdacaGLOaGaayzkaaaaaa@6D93@

Note that the denominator of (3) is the same as (1), and can therefore be calculated by Algorithm 1 above, while the numerator of (3) is calculated by Algorithm 2 below.

### Algorithm 2: derivative of local alignment kernel

Initialization:for  i=0,…,|x|  and  j=0,…,|y|:M′i,0=M′0,j=X′i,0=X′0,j=X2′i,0=X2′0,j=Y′i,0=Y′0,j=Y2′i,0=Y2′0,j=0,Iteration:for  i=1,…,|x|  and  j=1,…,|y|:M′i,j=∂∂S(a,b)Mi,j=δ((xi,yi)=(a,b))βeβS(xi,yi)(1+Mi−1,j−1+Xi−1,j−1+Yi−1,j−1)+eβS(xi,yi)(M′i−1,j−1+X′i−1,j−1+Y′i−1,j−1),X′i,j=∂∂S(a,b)Xi,j=eβgdM′i−1,j+eβgeX′i−1,j,Y′i,j=∂∂S(a,b)Yi,j=eβgd(M′i,j−1+X′i,j−1)+eβgeY′i,j−1,X2′i,j=∂∂S(a,b)X2i,j=M′i−1,j+X2′i−1,j,Y2′i,j=∂∂S(a,b)Y2i,j=M′i,j−1+X2′i,j−1+Y2′i,j−1,Termination:∂∂S(a,b)KLA(x,y)=M′|x|,|y|+X2′|x|,|y|+Y2′|x|,|y|.
 MathType@MTEF@5@5@+=feaafiart1ev1aaatCvAUfKttLearuWrP9MDH5MBPbIqV92AaeXatLxBI9gBaebbnrfifHhDYfgasaacH8akY=wiFfYdH8Gipec8Eeeu0xXdbba9frFj0=OqFfea0dXdd9vqai=hGuQ8kuc9pgc9s8qqaq=dirpe0xb9q8qiLsFr0=vr0=vr0dc8meaabaqaciaacaGaaeqabaqabeGadaaakeaafaqaaeWcdaaaaaqaceaaqlGaaCzcaiabbMeajjabb6gaUjabbMgaPjabbsha0jabbMgaPjabbggaHjabbYgaSjabbMgaPjabbQha6jabbggaHjabbsha0jabbMgaPjabb+gaVjabb6gaUbqaaiabbQda6aqaaiabbAgaMjabb+gaVjabbkhaYjabbccaGiabbccaGiabdMgaPjabg2da9iabicdaWiabcYcaSiablAciljabcYcaSmaaemaabaacbeGae8hEaGhacaGLhWUaayjcSdGaeeiiaaIaeeiiaaIaeeyyaeMaeeOBa4MaeeizaqMaeeiiaaIaeeiiaaIaemOAaOMaeyypa0JaeGimaaJaeiilaWIaeSOjGSKaeiilaWYaaqWaaeaacqWF5bqEaiaawEa7caGLiWoacqGG6aGoaeGabaaohiaaxMaacuWGnbqtgaqbamaaBaaaleaacqWGPbqAcqGGSaalcqaIWaamaeqaaaGcbaGaeyypa0dabaGafmyta0KbauaadaWgaaWcbaGaeGimaaJaeiilaWIaemOAaOgabeaakiabg2da9iqbdIfayzaafaWaaSbaaSqaaiabdMgaPjabcYcaSiabicdaWaqabaGccqGH9aqpcuWGybawgaqbamaaBaaaleaacqaIWaamcqGGSaalcqWGQbGAaeqaaOGaeyypa0JaemiwaGLafGOmaiJbauaadaWgaaWcbaGaemyAaKMaeiilaWIaeGimaadabeaakiabg2da9iabdIfayjqbikdaYyaafaWaaSbaaSqaaiabicdaWiabcYcaSiabdQgaQbqabaGccqGH9aqpcuWGzbqwgaqbamaaBaaaleaacqWGPbqAcqGGSaalcqaIWaamaeqaaOGaeyypa0JafmywaKLbauaadaWgaaWcbaGaeGimaaJaeiilaWIaemOAaOgabeaakiabg2da9iabdMfazjqbikdaYyaafaWaaSbaaSqaaiabdMgaPjabcYcaSiabicdaWaqabaGccqGH9aqpcqWGzbqwcuaIYaGmgaqbamaaBaaaleaacqaIWaamcqGGSaalcqWGQbGAaeqaaOGaeyypa0JaeGimaaJaeiilaWcabiqaaqqbcaWLjaGaeeysaKKaeeiDaqNaeeyzauMaeeOCaiNaeeyyaeMaeeiDaqNaeeyAaKMaee4Ba8MaeeOBa4gabaGaeeOoaOdabaGaeeOzayMaee4Ba8MaeeOCaiNaeeiiaaIaeeiiaaIaemyAaKMaeyypa0JaeGymaeJaeiilaWIaeSOjGSKaeiilaWYaaqWaaeaacqWF4baEaiaawEa7caGLiWoacqqGGaaicqqGGaaicqqGHbqycqqGUbGBcqqGKbazcqqGGaaicqqGGaaicqWGQbGAcqGH9aqpcqaIXaqmcqGGSaalcqWIMaYscqGGSaaldaabdaqaaiab=Lha5bGaay5bSlaawIa7aiabcQda6aqaceaaODGaaCzcaiqbd2eanzaafaWaaSbaaSqaaiabdMgaPjabcYcaSiabdQgaQbqabaaakeaacqGH9aqpaeaadaWcaaqaaiabgkGi2cqaaiabgkGi2kabdofatnaabmaabaGaemyyaeMaeiilaWIaemOyaigacaGLOaGaayzkaaaaaiabd2eannaaBaaaleaacqWGPbqAcqGGSaalcqWGQbGAaeqaaOGaeyypa0dcciGae4hTdq2aaSbaaSqaaiabcIcaOiabcIcaOiabdIha4naaBaaameaacqWGPbqAaeqaaSGaeiilaWIaemyEaK3aaSbaaWqaaiabdMgaPbqabaWccqGGPaqkcqGH9aqpcqGGOaakcqWGHbqycqGGSaalcqWGIbGycqGGPaqkcqGGPaqkaeqaaOGae4NSdiMaemyzau2aaWbaaSqabeaacqGFYoGycqWGtbWudaqadaqaaiabdIha4naaBaaameaacqWGPbqAaeqaaSGaeiilaWIaemyEaK3aaSbaaWqaaiabdMgaPbqabaaaliaawIcacaGLPaaaaaGcdaqadaqaaiabigdaXiabgUcaRiabd2eannaaBaaaleaacqWGPbqAcqGHsislcqaIXaqmcqGGSaalcqWGQbGAcqGHsislcqaIXaqmaeqaaOGaey4kaSIaemiwaG1aaSbaaSqaaiabdMgaPjabgkHiTiabigdaXiabcYcaSiabdQgaQjabgkHiTiabigdaXaqabaGccqGHRaWkcqWGzbqwdaWgaaWcbaGaemyAaKMaeyOeI0IaeGymaeJaeiilaWIaemOAaOMaeyOeI0IaeGymaedabeaaaOGaayjkaiaawMcaaaqaaaqaaiabgUcaRaqaaiabdwgaLnaaCaaaleqabaGae4NSdiMaem4uam1aaeWaaeaacqWG4baEdaWgaaadbaGaemyAaKgabeaaliabcYcaSiabdMha5naaBaaameaacqWGPbqAaeqaaaWccaGLOaGaayzkaaaaaOWaaeWaaeaacuWGnbqtgaqbamaaBaaaleaacqWGPbqAcqGHsislcqaIXaqmcqGGSaalcqWGQbGAcqGHsislcqaIXaqmaeqaaOGaey4kaSIafmiwaGLbauaadaWgaaWcbaGaemyAaKMaeyOeI0IaeGymaeJaeiilaWIaemOAaOMaeyOeI0IaeGymaedabeaakiabgUcaRiqbdMfazzaafaWaaSbaaSqaaiabdMgaPjabgkHiTiabigdaXiabcYcaSiabdQgaQjabgkHiTiabigdaXaqabaaakiaawIcacaGLPaaacqGGSaalaeGabaaShiaaxMaacuWGybawgaqbamaaBaaaleaacqWGPbqAcqGGSaalcqWGQbGAaeqaaaGcbaGaeyypa0dabaWaaSaaaeaacqGHciITaeaacqGHciITcqWGtbWudaqadaqaaiabdggaHjabcYcaSiabdkgaIbGaayjkaiaawMcaaaaacqWGybawdaWgaaWcbaGaemyAaKMaeiilaWIaemOAaOgabeaakiabg2da9iabdwgaLnaaCaaaleqabaGae4NSdiMaem4zaC2aaSbaaWqaaiabdsgaKbqabaaaaOGafmyta0KbauaadaWgaaWcbaGaemyAaKMaeyOeI0IaeGymaeJaeiilaWIaemOAaOgabeaakiabgUcaRiabdwgaLnaaCaaaleqabaGae4NSdiMaem4zaC2aaSbaaWqaaiabdwgaLbqabaaaaOGafmiwaGLbauaadaWgaaWcbaGaemyAaKMaeyOeI0IaeGymaeJaeiilaWIaemOAaOgabeaakiabcYcaSaqaceaauFGaaCzcaiqbdMfazzaafaWaaSbaaSqaaiabdMgaPjabcYcaSiabdQgaQbqabaaakeaacqGH9aqpaeaadaWcaaqaaiabgkGi2cqaaiabgkGi2kabdofatnaabmaabaGaemyyaeMaeiilaWIaemOyaigacaGLOaGaayzkaaaaaiabdMfaznaaBaaaleaacqWGPbqAcqGGSaalcqWGQbGAaeqaaOGaeyypa0Jaemyzau2aaWbaaSqabeaacqGFYoGycqWGNbWzdaWgaaadbaGaemizaqgabeaaaaGcdaqadaqaaiqbd2eanzaafaWaaSbaaSqaaiabdMgaPjabcYcaSiabdQgaQjabgkHiTiabigdaXaqabaGccqGHRaWkcuWGybawgaqbamaaBaaaleaacqWGPbqAcqGGSaalcqWGQbGAcqGHsislcqaIXaqmaeqaaaGccaGLOaGaayzkaaGaey4kaSIaemyzau2aaWbaaSqabeaacqGFYoGycqWGNbWzdaWgaaadbaGaemyzaugabeaaaaGccuWGzbqwgaqbamaaBaaaleaacqWGPbqAcqGGSaalcqWGQbGAcqGHsislcqaIXaqmaeqaaOGaeiilaWcabiqaaWVbcaWLjaGaemiwaGLafGOmaiJbauaadaWgaaWcbaGaemyAaKMaeiilaWIaemOAaOgabeaaaOqaaiabg2da9aqaamaalaaabaGaeyOaIylabaGaeyOaIyRaem4uam1aaeWaaeaacqWGHbqycqGGSaalcqWGIbGyaiaawIcacaGLPaaaaaGaemiwaGLaeGOmaiZaaSbaaSqaaiabdMgaPjabcYcaSiabdQgaQbqabaGccqGH9aqpcuWGnbqtgaqbamaaBaaaleaacqWGPbqAcqGHsislcqaIXaqmcqGGSaalcqWGQbGAaeqaaOGaey4kaSIaemiwaGLafGOmaiJbauaadaWgaaWcbaGaemyAaKMaeyOeI0IaeGymaeJaeiilaWIaemOAaOgabeaakiabcYcaSaqaceaaGCGaaCzcaiabdMfazjqbikdaYyaafaWaaSbaaSqaaiabdMgaPjabcYcaSiabdQgaQbqabaaakeaacqGH9aqpaeaadaWcaaqaaiabgkGi2cqaaiabgkGi2kabdofatnaabmaabaGaemyyaeMaeiilaWIaemOyaigacaGLOaGaayzkaaaaaiabdMfazjabikdaYmaaBaaaleaacqWGPbqAcqGGSaalcqWGQbGAaeqaaOGaeyypa0Jafmyta0KbauaadaWgaaWcbaGaemyAaKMaeiilaWIaemOAaOMaeyOeI0IaeGymaedabeaakiabgUcaRiabdIfayjqbikdaYyaafaWaaSbaaSqaaiabdMgaPjabcYcaSiabdQgaQjabgkHiTiabigdaXaqabaGccqGHRaWkcqWGzbqwcuaIYaGmgaqbamaaBaaaleaacqWGPbqAcqGGSaalcqWGQbGAcqGHsislcqaIXaqmaeqaaOGaeiilaWcabiqaaaWacaWLjaGaeeivaqLaeeyzauMaeeOCaiNaeeyBa0MaeeyAaKMaeeOBa4MaeeyyaeMaeeiDaqNaeeyAaKMaee4Ba8MaeeOBa4gabaGaeeOoaOdabaaabaWaaSaaaeaacqGHciITaeaacqGHciITcqWGtbWudaqadaqaaiabdggaHjabcYcaSiabdkgaIbGaayjkaiaawMcaaaaacqWGlbWsdaWgaaWcbaGaemitaWKaemyqaeeabeaakmaabmaabaGae8hEaGNae8hlaWIae8xEaKhacaGLOaGaayzkaaaabaGaeyypa0dabaGafmyta0KbauaadaWgaaWcbaWaaqWaaeaacqWF4baEaiaawEa7caGLiWoacqGGSaaldaabdaqaaiab=Lha5bGaay5bSlaawIa7aaqabaGccqGHRaWkcqWGybawcuaIYaGmgaqbamaaBaaaleaadaabdaqaaiab=Hha4bGaay5bSlaawIa7aiabcYcaSmaaemaabaGae8xEaKhacaGLhWUaayjcSdaabeaakiabgUcaRiabdMfazjqbikdaYyaafaWaaSbaaSqaamaaemaabaGae8hEaGhacaGLhWUaayjcSdGaeiilaWYaaqWaaeaacqWF5bqEaiaawEa7caGLiWoaaeqaaOGaeiOla4caaaaa@6ADC@

In the above algorithm, *δ*((*x*_*i*_, *y*_*j*_) * = (a, b)) *is the Kronecker delta function which returns one if the *i*th amino acid of **x **is *a *and the *j*th amino acid of **y **is *b*, and zero otherwise. Derivative of local alignment kernel with respect to the gap open parameter *g*_*d *_and gap extension parameter *g*_*e *_can be calculated similarly.

### Objective function

To assess the significance of the score on a database search, the Z-score is widely used:

Z=s−μ〈s2〉−μ2=s−μσ,
 MathType@MTEF@5@5@+=feaafiart1ev1aaatCvAUfKttLearuWrP9MDH5MBPbIqV92AaeXatLxBI9gBaebbnrfifHhDYfgasaacH8akY=wiFfYdH8Gipec8Eeeu0xXdbba9frFj0=OqFfea0dXdd9vqai=hGuQ8kuc9pgc9s8qqaq=dirpe0xb9q8qiLsFr0=vr0=vr0dc8meaabaqaciaacaGaaeqabaqabeGadaaakeaacqWGAbGwcqGH9aqpdaWcaaqaaiabdohaZjabgkHiTGGaciab=X7aTbqaamaakaaabaWaaaWaaeaacqWGZbWCdaahaaWcbeqaaiabikdaYaaaaOGaayzkJiaawQYiaaWcbeaakiabgkHiTiab=X7aTnaaCaaaleqabaGaeGOmaidaaaaakiabg2da9maalaaabaGaem4CamNaeyOeI0Iae8hVd0gabaGae83WdmhaaiabcYcaSaaa@432D@

where *s *is the score of a query against a candidate homolog in the database, and *μ *and σ are the mean and variance of the scores of a query versus possible non-homologs in the database. For extreme values (maxima or minima) such as the SW score, the extreme value distribution (EVD) is commonly used to assess the statistical significance of the scores. The probability that a given random score is equal to or greater than *s *is given by

p(μ>s)=1−e−e−aZ−b,
 MathType@MTEF@5@5@+=feaafiart1ev1aaatCvAUfKttLearuWrP9MDH5MBPbIqV92AaeXatLxBI9gBaebbnrfifHhDYfgasaacH8akY=wiFfYdH8Gipec8Eeeu0xXdbba9frFj0=OqFfea0dXdd9vqai=hGuQ8kuc9pgc9s8qqaq=dirpe0xb9q8qiLsFr0=vr0=vr0dc8meaabaqaciaacaGaaeqabaqabeGadaaakeaacqWGWbaCdaqadaqaaGGaciab=X7aTjabg6da+iabdohaZbGaayjkaiaawMcaaiabg2da9iabigdaXiabgkHiTiabdwgaLnaaCaaaleqabaGaeyOeI0Iaemyzau2aaWbaaWqabeaacqGHsislcqWGHbqycqWGAbGwcqGHsislcqWGIbGyaaaaaOGaeiilaWcaaa@413C@

where *a *and *b *are parameters for the extreme value distribution. Then for the search of a database of size *D*, the expected number of scores which are higher than *s *is *E = Dp(μ *> *s*). A natural objective function to quantify the performance of an algorithm for remote homology is therefore to minimize the E-values obtained on pairs of distant homologs. Following Kann et al. [19], we consider the confidence value *C = *1/(1 + *E*), setting *D *= 100000 for the computation of the *E-*value, and define our objective function to be maximized as the average of *C *over a training set of homologous pairs.

If the score *s *is differentiable with respect to an amino acid substitution matrix and gap penalties (which we denote as a parameter *θ *here), then *C *and the derivative of *C *can be written as:

C=(1+D(1−e−e−αZ−β))−1,     (4)∂C∂θ=αC2De−aZ−b−e−aZ−b∂Z∂θ.
 MathType@MTEF@5@5@+=feaafiart1ev1aaatCvAUfKttLearuWrP9MDH5MBPbIqV92AaeXatLxBI9gBaebbnrfifHhDYfgasaacH8akY=wiFfYdH8Gipec8Eeeu0xXdbba9frFj0=OqFfea0dXdd9vqai=hGuQ8kuc9pgc9s8qqaq=dirpe0xb9q8qiLsFr0=vr0=vr0dc8meaabaqaciaacaGaaeqabaqabeGadaaakeaafaqaaeGadaaabiGaaW9=aqlacaWLjaGaem4qameabaGaeyypa0dabaWaaeWaaeaacqaIXaqmcqGHRaWkcqWGebarcqGGOaakcqaIXaqmcqGHsislcqWGLbqzdaahaaWcbeqaaiabgkHiTiabdwgaLnaaCaaameqabaGaeyOeI0ccciGae8xSdeMaemOwaOLaeyOeI0Iae8NSdigaaaaakiabcMcaPaGaayjkaiaawMcaamaaCaaaleqabaGaeyOeI0IaeGymaedaaOGaeiilaWIaaCzcaiaaxMaadaqadaqaaiabisda0aGaayjkaiaawMcaaaqaamaalaaabaGaeyOaIyRaem4qameabaGaeyOaIyRae8hUdehaaaqaaiabg2da9aqaaiab=f7aHjabdoeadnaaCaaaleqabaGaeGOmaidaaOGaemiraqKaemyzau2aaWbaaSqabeaacqGHsislcqWGHbqycqWGAbGwcqGHsislcqWGIbGycqGHsislcqWGLbqzdaahaaadbeqaaiabgkHiTiabdggaHjabdQfaAjabgkHiTiabdkgaIbaaaaGcdaWcaaqaaiabgkGi2kabdQfaAbqaaiabgkGi2kab=H7aXbaacqGGUaGlaaaaaa@6C94@

The derivative of *Z *with respect to *θ *is itself obtained by:

∂Z∂θ=1σ2[σ(∂s∂θ−〈∂s∂θ〉)−s−μσ(〈s∂s∂θ〉−μ〈∂s∂θ〉)].
 MathType@MTEF@5@5@+=feaafiart1ev1aaatCvAUfKttLearuWrP9MDH5MBPbIqV92AaeXatLxBI9gBaebbnrfifHhDYfgasaacH8akY=wiFfYdH8Gipec8Eeeu0xXdbba9frFj0=OqFfea0dXdd9vqai=hGuQ8kuc9pgc9s8qqaq=dirpe0xb9q8qiLsFr0=vr0=vr0dc8meaabaqaciaacaGaaeqabaqabeGadaaakeaadaWcaaqaaiabgkGi2kabdQfaAbqaaiabgkGi2IGaciab=H7aXbaacqGH9aqpdaWcaaqaaiabigdaXaqaaiab=n8aZnaaCaaaleqabaGae8NmaidaaaaakmaadmaabaGae83Wdm3aaeWaaeaadaWcaaqaaiabgkGi2kabdohaZbqaaiabgkGi2kab=H7aXbaacqGHsisldaaadaqaamaalaaabaGaeyOaIyRaem4CamhabaGaeyOaIyRae8hUdehaaaGaayzkJiaawQYiaaGaayjkaiaawMcaaiabgkHiTmaalaaabaGaem4CamNaeyOeI0Iae8hVd0gabaGae83WdmhaamaabmaabaWaaaWaaeaacqWGZbWCdaWcaaqaaiabgkGi2kabdohaZbqaaiabgkGi2kab=H7aXbaaaiaawMYicaGLQmcacqGHsislcqWF8oqBdaaadaqaamaalaaabaGaeyOaIyRaem4CamhabaGaeyOaIyRae8hUdehaaaGaayzkJiaawQYiaaGaayjkaiaawMcaaaGaay5waiaaw2faaiabc6caUaaa@682E@

**Figure 8:**
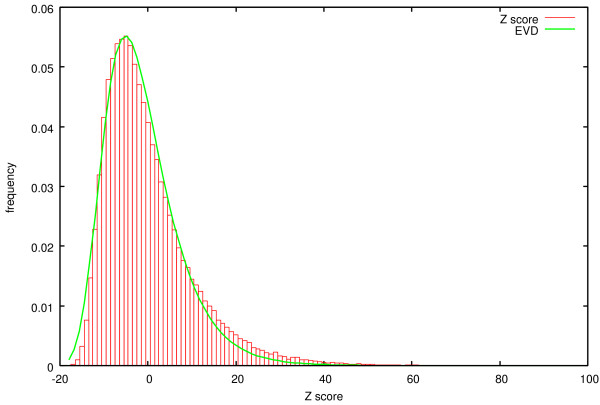
**Distribution of Z score of the LA kernel**. The curve shows that the Z scores of the LA kernel of non-homologous sequences of the same length randomly shuffled 100000 times follow the Extreme Value Distribution (e((a−x)/b−e((a−x)/b))/b
 MathType@MTEF@5@5@+=feaafiart1ev1aaatCvAUfKttLearuWrP9MDH5MBPbIqV92AaeXatLxBI9gBaebbnrfifHhDYfgasaacH8akY=wiFfYdH8Gipec8Eeeu0xXdbba9frFj0=OqFfea0dXdd9vqai=hGuQ8kuc9pgc9s8qqaq=dirpe0xb9q8qiLsFr0=vr0=vr0dc8meaabaqaciaacaGaaeqabaqabeGadaaakeaacqGGOaakcqWGLbqzdaahaaWcbeqaaiabcIcaOiabcIcaOiabdggaHjabgkHiTiabdIha4jabcMcaPiabc+caViabdkgaIjabgkHiTiabdwgaLnaaCaaameqabaGaeiikaGIaeiikaGIaemyyaeMaeyOeI0IaemiEaGNaeiykaKIaei4la8IaemOyaiMaeiykaKcaaSGaeiykaKcaaOGaei4la8IaemOyaigaaa@464A@ with a = -5.1, b = 6.7).

Concerning the validity of assuming that the score of the LA kernel follows an extreme value distribution, we randomly shuffled non-homologous sequence of the same length 100000 times, and observed that the extreme value distribution is still a good approximation for the distribution of scores of the LA kernel (Figure 8). We chose *β *= 0.5 obtained from previous research, i.e., by moving *β *from 0 to 1 with an interval of 0.1 and choosing the best performing *β *[7]. In fact, we can run gradient descent with respect to the matrix and *β *together. But the point is that the system is over-parameterized, and fixing *β *= 0.5 will have no influence at the end.

### Optimization procedure

We used both the algorithms of Smith-Waterman and the LA kernel together with their derivatives, then maximized the objective function using gradient descent with Armijo's rule for line search [21].

For the optimization using Smith-Waterman algorithm, we adopted the same method as in [19], that is, to alternate both local optimization of the substitution matrix by gradient descent and computation of the best scoring alignment. Note that this alternation is not necessary the LA kernel.

Since there is no guarantee of reaching the global optimum, we used several starting matrices such as BLOSUM62, PAM250, GCB and JTT, with default gap parameters (12 and 2 for open and extension, respectively). In order to limit the over-fitting of the parameters to the training set, the optimization was carried out until the objective function reached a maximum on an independent validation set. The performance of the parameters selected by this procedure was then assessed on an independent test set.

### Relationship between the LA kernel and the SW algorithm

It is known that the LA kernel is an approximation of the SW score for large *β *[7]. More precisely, the following holds:

lim⁡β→+∞1βK^LA(β)(x,y)=SW(x,y).
 MathType@MTEF@5@5@+=feaafiart1ev1aaatCvAUfKttLearuWrP9MDH5MBPbIqV92AaeXatLxBI9gBaebbnrfifHhDYfgasaacH8akY=wiFfYdH8Gipec8Eeeu0xXdbba9frFj0=OqFfea0dXdd9vqai=hGuQ8kuc9pgc9s8qqaq=dirpe0xb9q8qiLsFr0=vr0=vr0dc8meaabaqaciaacaGaaeqabaqabeGadaaakeaadaWfqaqaaiGbcYgaSjabcMgaPjabc2gaTbWcbaacciGae8NSdiMaeyOKH4Qaey4kaSIaeyOhIukabeaakmaalaaabaGaeGymaedabaGae8NSdigaaiqbdUealzaajaWaa0baaSqaaiabdYeamjabdgeabbqaamaabmaabaGae8NSdigacaGLOaGaayzkaaaaaOWaaeWaaeaaieqacqGF4baEcqGGSaalcqGF5bqEaiaawIcacaGLPaaacqGH9aqpcqWGtbWucqWGxbWvdaqadaqaaiab+Hha4jabcYcaSiab+Lha5bGaayjkaiaawMcaaiabc6caUaaa@4F48@

Furthermore, the derivative of the LA kernel (1) with respect to the substitution score *S(a, b) *is equal to:

∂K^LA(β)(x,y)∂S(a,b)=∑πβna,b(x,y,π)eβs(x,y,π)∑πeβs(x,y,π),     (5)=Eπ[βna,b(x,y,π)],     (6)
 MathType@MTEF@5@5@+=feaafiart1ev1aaatCvAUfKttLearuWrP9MDH5MBPbIqV92AaeXatLxBI9gBaebbnrfifHhDYfgasaacH8akY=wiFfYdH8Gipec8Eeeu0xXdbba9frFj0=OqFfea0dXdd9vqai=hGuQ8kuc9pgc9s8qqaq=dirpe0xb9q8qiLsFr0=vr0=vr0dc8meaabaqaciaacaGaaeqabaqabeGadaaakeaafaqadeGacaaabaWaaSaaaeaacqGHciITcuWGlbWsgaqcamaaDaaaleaacqWGmbatcqWGbbqqaeaadaqadaqaaGGaciab=j7aIbGaayjkaiaawMcaaaaakmaabmaabaacbeGae4hEaGNaeiilaWIae4xEaKhacaGLOaGaayzkaaaabaGaeyOaIyRaem4uam1aaeWaaeaacqWGHbqycqGGSaalcqWGIbGyaiaawIcacaGLPaaaaaGaeyypa0ZaaSaaaeaadaaeqaqaaiab=j7aIjabd6gaUnaaBaaaleaacqWGHbqycqGGSaalcqWGIbGyaeqaaOWaaeWaaeaacqGF4baEcqGGSaalcqGF5bqEcqGFSaalcqWFapaCaiaawIcacaGLPaaaaSqaaiab=b8aWbqab0GaeyyeIuoakiabdwgaLnaaCaaaleqabaGae8NSdiMaem4Cam3aaeWaaeaacqGF4baEcqGGSaalcqGF5bqEcqGFSaalcqWFapaCaiaawIcacaGLPaaaaaaakeaadaaeqaqaaiabdwgaLnaaCaaaleqabaGae8NSdiMaem4Cam3aaeWaaeaacqGF4baEcqGGSaalcqGF5bqEcqGFSaalcqWFapaCaiaawIcacaGLPaaaaaaabaGae8hWdahabeqdcqGHris5aaaakiabcYcaSaqaaiaaxMaacaWLjaWaaeWaaeaacqaI1aqnaiaawIcacaGLPaaaaeaacqGH9aqpcqWGfbqrdaWgaaWcbaGae8hWdahabeaakiabcUfaBjab=j7aIjabd6gaUnaaBaaaleaacqWGHbqycqGGSaalcqWGIbGyaeqaaOGaeiikaGIae4hEaGNaeiilaWIae4xEaKNae4hlaWIae8hWdaNaeiykaKIaeiyxa0LaeiilaWcabaGaaCzcaiaaxMaadaqadaqaaiabiAda2aGaayjkaiaawMcaaaaaaaa@9055@

where *E*_*π *_denotes expectation with respect to the probability distribution p(π)=eβs(x,y,π)∑πeβs(x,y,π)
 MathType@MTEF@5@5@+=feaafiart1ev1aaatCvAUfKttLearuWrP9MDH5MBPbIqV92AaeXatLxBI9gBaebbnrfifHhDYfgasaacH8akY=wiFfYdH8Gipec8Eeeu0xXdbba9frFj0=OqFfea0dXdd9vqai=hGuQ8kuc9pgc9s8qqaq=dirpe0xb9q8qiLsFr0=vr0=vr0dc8meaabaqaciaacaGaaeqabaqabeGadaaakeaacqWGWbaCdaqadaqaaGGaciab=b8aWbGaayjkaiaawMcaaiabg2da9maalaaabaGaemyzau2aaWbaaSqabeaacqWFYoGycqWGZbWCdaqadaqaaGqabiab+Hha4jabcYcaSiab+Lha5jabcYcaSiab=b8aWbGaayjkaiaawMcaaaaaaOqaamaaqababaGaemyzau2aaWbaaSqabeaacqWFYoGycqWGZbWCdaqadaqaaiab+Hha4jabcYcaSiab+Lha5jabcYcaSiab=b8aWbGaayjkaiaawMcaaaaaaeaacqWFapaCaeqaniabggHiLdaaaaaa@4F08@ on the set of possible alignments *π *. The probability of an alignment *π *therefore contributes to a proportion of the score of an alignment *π *to the score K^LA(β)(x,y)
 MathType@MTEF@5@5@+=feaafiart1ev1aaatCvAUfKttLearuWrP9MDH5MBPbIqV92AaeXatLxBI9gBaebbnrfifHhDYfgasaacH8akY=wiFfYdH8Gipec8Eeeu0xXdbba9frFj0=OqFfea0dXdd9vqai=hGuQ8kuc9pgc9s8qqaq=dirpe0xb9q8qiLsFr0=vr0=vr0dc8meaabaqaciaacaGaaeqabaqabeGadaaakeaacuWGlbWsgaqcamaaDaaaleaacqWGmbatcqWGbbqqaeaadaqadaqaaGGaciab=j7aIbGaayjkaiaawMcaaaaakmaabmaabaacbeGae4hEaGNaeiilaWIae4xEaKhacaGLOaGaayzkaaaaaa@38CC@, and forms a Gibbs distribution over tt with energy –*s(x, y, π). β *can be thought of as the inverse temperature: at low temperature (large *β*), only the low-energy states (large score) have non-vanishing probability; at large temperature (small *β*), all states (all scores) have similar probability. Denoting by Π_0_(**x**, **y**) the set of alignments *π *that have the maximum score, this shows that at low temperature one gets:

lim⁡β→+∞1β∂K^LA(β)(x,y)∂S(a,b)=1|Π0(x,y)|∑π∈Π0(x,y)na,b(x,y,π).
 MathType@MTEF@5@5@+=feaafiart1ev1aaatCvAUfKttLearuWrP9MDH5MBPbIqV92AaeXatLxBI9gBaebbnrfifHhDYfgasaacH8akY=wiFfYdH8Gipec8Eeeu0xXdbba9frFj0=OqFfea0dXdd9vqai=hGuQ8kuc9pgc9s8qqaq=dirpe0xb9q8qiLsFr0=vr0=vr0dc8meaabaqaciaacaGaaeqabaqabeGadaaakeaadaWfqaqaaiGbcYgaSjabcMgaPjabc2gaTbWcbaacciGae8NSdiMaeyOKH4Qaey4kaSIaeyOhIukabeaakmaalaaabaGaeGymaedabaGae8NSdigaamaalaaabaGaeyOaIyRafm4saSKbaKaadaqhaaWcbaGaemitaWKaemyqaeeabaWaaeWaaeaacqWFYoGyaiaawIcacaGLPaaaaaGcdaqadaqaaGqabiab+Hha4jabcYcaSiab+Lha5bGaayjkaiaawMcaaaqaaiabgkGi2kabdofatnaabmaabaGaemyyaeMaeiilaWIaemOyaigacaGLOaGaayzkaaaaaiabg2da9maalaaabaGaeGymaedabaWaaqWaaeaacqqHGoaudaWgaaWcbaGaeGimaadabeaakmaabmaabaGae4hEaGNaeiilaWIae4xEaKhacaGLOaGaayzkaaaacaGLhWUaayjcSdaaamaaqafabaGaemOBa42aaSbaaSqaaiabdggaHjabcYcaSiabdkgaIbqabaGcdaqadaqaaiab+Hha4jabcYcaSiab+Lha5jab+XcaSiab=b8aWbGaayjkaiaawMcaaaWcbaGae8hWdaNaeyicI4SaeuiOda1aaSbaaWqaaiabicdaWaqabaWcdaqadaqaaiab+Hha4jabcYcaSiab+Lha5bGaayjkaiaawMcaaaqab0GaeyyeIuoakiabc6caUaaa@770C@

In the case where there exists a single alignment *π*_0 _that maximizes the score, then this reduces to *n*_*a*,*b*_(**x**, **y**, *π*_0_), which is exactly the derivative of the SW score in this case. This result clarifies the difference between taking the derivative of the LA kernel and that of the SW score when it exists. The derivative of the SW score is the amino acid residue count in the optimal alignment, while the derivative of the LA kernel is an expectation of an amino acid residue count over possible alignments. As a result, up to the *β *factor, the derivative of the LA kernel is an approximation of the derivative of the SW score when it exists. In particular the gradient of the LA kernel approximates the gradient used in the parameter optimization step of Kann et al.'s algorithm for large *β*. The same approximation properties hold for higher-order differentials, although the LA kernel is everywhere infinitely differentiable while the SW score is only piecewise linear over the space of substitution matrices.

### Dataset

Training and testing to discriminate homologs from non-homologs was performed on the Cluster of Orthologous Group (COG) database [6]. We were interested in homologs whose homology is hard to detect, and collected sequences with less than 20% identity only from the COG database, resulting in 395 pairs of protein sequences. We used 300 of them for training, 48 for validation and the rest (47) for evaluation. Note that this threshold of identity (20%) is harder than that of Kann et al.'s methods in order to learn known but clearly distant relationships of homologs. Also, since it is always important to assess the confidence in an independent way, we prepared sequence pairs of distant homologs from the PFAM [5] database in a similar manner, resulting in 200 additional pairs, and used them as the second test set. The third and the fourth data sets are the COG close and PFAM close datasets – prepared by keeping the identity between 25% and 40%. We ran SSEARCH on all the sequences in the PFAM database against all the training and test set sequences from COGs in order to remove the similar sequences from the PFAM dataset using a threshold of *E <*10.

## Authors' contributions

HS extended the code, carried out experiments and drafted the manuscript. JPV conceived of the study and did the original coding. TA provided discussion on the methodology and algorithm. JPV and TA participated in discussion and preparation of manuscript. All three authors read and approved the final manuscript.

## Figures and Tables

**Table 1:** Amino acid substitution matrix optimized for the SW score. Optimization was started from the BLOSUM62 matrix with gap open and extension penalties initialized to 12 and 2 respectively, on COG training data. After the optimization procedure, the open and extension penalties are 12.3 and 2.8, respectively.

A	4.5																			
R	-0.9	5.2																		
N	-1.9	0.1	5.9																	
D	-2.0	-2.0	1.2	6.2																
C	0.1	-3.0	-3.0	-3.0	9.0															
Q	-0.9	1.0	0.0	0.1	-3.0	5.1														
E	-0.8	0.1	0.1	2.1	-4.0	2.1	5.0													
G	0.2	-2.0	0.1	-0.9	-3.0	-1.9	-1.8	6.3												
H	-2.0	0.0	1.1	-1.0	-3.0	-0.0	0.1	-2.0	8.0											
I	-0.8	-2.9	-3.0	-3.1	-1.0	-3.0	-3.1	-3.9	-3.0	4.0										
L	-0.8	-1.9	-3.0	-4.0	-1.0	-2.0	-2.9	-3.9	-3.0	2.6	4.4									
K	-0.8	2.3	0.0	-1.0	-3.0	1.2	1.1	-2.0	-0.9	-3.0	-1.9	4.8								
M	-1.0	-1.0	-2.0	-3.0	-1.0	0.0	-2.0	-3.0	-2.0	1.2	2.1	-1.0	5.0							
F	-1.9	-2.9	-3.0	-3.0	-2.0	-3.0	-3.0	-3.0	-1.0	0.2	0.3	-3.0	0.0	6.1						
P	-1.0	-2.0	-2.0	-0.9	-3.0	-1.0	-1.0	-1.9	-2.0	-3.0	-2.9	-1.0	-2.0	-4.0	7.1					
S	1.3	-1.0	1.1	0.1	-1.0	0.1	0.1	0.2	-1.0	-1.9	-1.9	-0.0	-1.0	-1.9	-0.9	4.0				
T	0.2	-1.0	0.1	-1.0	-1.0	-0.9	-0.9	-1.9	-2.0	-0.9	-0.8	-1.0	-1.0	-1.9	-1.0	1.1	5.1			
W	-3.0	-3.0	-4.0	-4.0	-2.0	-2.0	-3.0	-2.0	-2.0	-3.0	-1.9	-3.0	-1.0	1.0	-4.0	-3.0	-2.0	11.1		
Y	-1.9	-1.9	-2.0	-3.0	-2.0	-0.9	-2.0	-3.0	2.1	-1.0	-0.8	-2.0	-1.0	3.2	-3.0	-2.0	-2.0	2.0	7.0	
V	0.2	-3.0	-3.0	-3.0	-1.0	-2.0	-2.0	-3.0	-3.0	3.4	1.2	-2.0	1.1	-0.9	-1.9	-1.9	0.1	-3.0	-1.0	4.2
	A	R	N	D	C	Q	E	G	H	I	L	K	M	F	P	S	T	W	Y	V

**Table 2:** Amino acid substitution matrix optimized for the LA kernel (β = 0:5). Optimization was started from the BLOSUM62 matrix with gap open and extension penalties initialized to 12 and 2 respectively, on COG training data. After the optimization procedure, the gap open and extension penalties are 12.5 and 5.0, respectively.

A	3.7																			
R	-0.5	5.5																		
N	-1.6	0.2	5.6																	
D	-2.1	-2.2	1.9	6.2																
C	0.4	-2.9	-3.0	-2.9	9.1															
Q	-0.7	1.1	0.2	0.2	-2.9	5.2														
E	-0.2	1.0	0.0	2.6	-3.8	2.0	4.2													
G	0.3	-1.9	0.2	-0.6	-2.9	-1.8	-1.6	7.4												
H	-2.0	0.2	1.0	-0.6	-3.0	0.2	0.0	-1.8	7.9											
I	-0.4	-2.8	-2.8	-3.4	-0.9	-2.8	-3.5	-3.7	-3.1	3.5										
L	-1.5	-1.7	-3.1	-4.1	-0.7	-1.9	-2.1	-4.1	-2.7	2.8	4.0									
K	-0.5	3.7	-0.4	-0.9	-2.9	1.8	1.1	-1.8	-0.8	-3.0	-1.9	3.6								
M	-0.5	-0.8	-2.4	-2.8	-0.9	0.1	-2.2	-3.0	-1.9	1.4	2.2	-0.7	4.8							
F	-1.9	-2.9	-3.1	-3.4	-1.9	-3.1	-3.0	-2.7	-1.1	0.4	1.4	-2.7	-0.4	6.0						
P	-1.0	-1.9	-2.0	-1.2	-3.1	-1.0	-1.2	-1.7	-2.0	-3.3	-3.0	-1.0	-2.0	-4.2	7.2					
S	1.4	-0.9	1.1	0.1	-0.9	0.5	-0.4	0.4	-1.0	-2.3	-2.2	0.1	-0.9	-1.8	-0.7	3.4				
T	0.1	-1.0	-0.3	-1.1	-0.8	-0.6	-0.3	-1.7	-1.9	-1.2	-0.7	-1.2	-1.3	-1.8	-0.8	0.7	4.4			
W	-3.0	-2.9	-4.4	-3.8	-2.0	-1.9	-3.0	-2.0	-1.9	-2.8	-1.7	-3.1	-1.2	1.3	-4.0	-3.0	-2.0	11.2		
Y	-1.9	-1.9	-1.9	-3.0	-2.0	-1.0	-1.6	-3.0	2.5	-0.7	-0.7	-2.0	-0.9	2.9	-3.0	-1.8	-2.0	2.0	7.2	
V	-0.1	-2.8	-3.1	-3.6	-0.8	-1.9	-2.3	-3.1	-2.7	3.7	1.6	-2.1	0.9	-0.8	-1.9	-1.9	-0.3	-3.1	-1.3	3.4
	A	R	N	D	C	Q	E	G	H	I	L	K	M	F	P	S	T	W	Y	V

**Table 3:** Final amino acid substitution matrix optimized for the LA kernel (β = 0:5). Optimization was started from the BLOSUM62 matrix with gap open and extension penalties initialized to 12 and 2 using all the COG distant data. After the optimization procedure, the gap open and extension penalties are 11.6 and 5.7, respectively.

A	3.1																			
R	-1.4	4.5																		
N	-1.5	0.1	5.9																	
D	-1.8	-1.8	1.9	6.0																
C	0.3	-2.9	-3.0	-3.0	8.7															
Q	-0.5	1.5	0.4	-0.4	-3.1	4.7														
E	-1.3	0.2	-0.3	2.3	-4.0	2.1	3.2													
G	0.6	-2.6	0.6	-0.5	-3.0	-1.9	-1.7	6.9												
H	-2.0	0.3	0.8	-0.9	-3.0	0.3	-0.0	-1.7	8.1											
I	-1.5	-3.0	-3.3	-3.3	-0.8	-2.6	-3.4	-3.9	-3.0	3.5										
L	-0.5	-2.3	-3.3	-4.4	-1.2	-1.9	-2.6	-4.0	-2.9	2.7	3.6									
K	-1.0	3.7	0.5	-0.3	-3.1	0.8	0.0	-1.5	-0.7	-3.0	-1.9	4.0								
M	-0.5	-0.9	-2.1	-3.0	-0.9	0.3	-2.2	-3.1	-2.0	1.5	2.5	-1.3	4.8							
F	-1.3	-2.5	-3.0	-2.8	-1.9	-2.9	-3.3	-3.2	-1.2	0.3	1.0	-3.0	0.2	5.2						
P	-1.0	-2.3	-1.9	-0.4	-3.1	-1.2	-1.2	-2.1	-2.0	-3.1	-2.5	-1.0	-1.9	-4.1	7.7					
S	2.2	-0.9	0.6	-0.1	-1.0	0.8	0.0	0.5	-0.8	-1.9	-2.4	0.1	-1.4	-1.8	-0.7	4.0				
T	1.0	-0.5	0.0	-0.7	-1.2	-0.7	-0.7	-1.9	-1.8	-1.3	-1.2	-0.7	-1.0	-1.6	-0.9	1.5	5.0			
W	-3.1	-3.0	-4.1	-4.0	-1.9	-1.9	-2.9	-1.9	-1.9	-2.8	-1.8	-2.9	-1.2	0.7	-4.0	-3.1	-2.0	10.5		
Y	-1.8	-1.6	-1.8	-3.1	-2.0	-1.0	-2.2	-3.5	2.3	-1.0	-0.9	-2.4	-0.8	2.8	-3.3	-1.9	-2.0	2.4	6.3	
V	0.5	-2.8	-2.6	-3.7	-0.7	-1.9	-1.9	-3.1	-3.1	4.0	1.2	-2.1	1.4	-0.4	-1.9	-2.3	0.2	-2.8	-1.0	3.9
	A	R	N	D	C	Q	E	G	H	I	L	K	M	F	P	S	T	W	Y	V

**Table 4:** ROC scores for the SW algorithm and the LA kernel in the independent dataset. The first column shows the scoring method. For example, BLOSUM62SWOPT is the matrix optimized for the SW algorithm starting from the BLOSUM62. The second column shows the performance of each score matrix by the SW algorithm on the COG distant test set. The following columns show the performance, in terms of average ROC score, of each matrix used in combination with either the SW algorithm or the LA kernel on four different datasets. The second row shows the performance of PSI-BLAST with the BLOSUM62 with gap open and extension parameters set to 11 and 1 (default), respectively. The best ROC score in each dataset is highlighted in bold font.

ROC score								
Method	COG distant		COG close		PFAM distant		PFAM close	
	SW	LA	SW	LA	SW	LA	SW	LA
PSI-BLAST	0.811		**0.953**		0.854		0.979	
BLOSUM62	0.840	0.852	0.950	0.951	0.931	0.932	0.985	**0.990**
BLOSUM62SWOPT	0.856	0.869	0.950	0.950	0.941	0.940	0.983	0.983
BLOSUM62LAOPT	0.878	**0.895**	0.949	0.948	0.946	**0.947**	0.984	0.982

**Table 5:** Comparison of various scoring matrices and scoring algorithms. The first column shows the scoring matrices. For example, BLOSUM62SWOPT is the matrix optimized for the SW algorithm starting from the BLOSUM62 matrix. The second column shows the performance of each score matrix by the SW algorithm on the COG distant test set. The following columns show the performance, in terms of average *C*, of each matrix used in combination with either the SW algorithm or the LA kernel on four different datasets. The best ⟨C⟩ in each column is highlighted in bold font.

⟨*C*⟩								
Score matrix	COG distant		COG close		PFAM distant		PFAM close	
	SW	LA	SW	LA	SW	LA	SW	LA
BLOSUM62	0.35	0.42	**0.72**	**0.73**	0.45	0.49	**0.76**	**0.78**
BLOSUM62SWOPT	0.42	0.42	0.70	0.71	**0.49**	0.51	0.75	0.77
BLOSUM62LAOPT	**0.43**	**0.51**	0.67	0.69	0.47	**0.54**	0.72	0.75
PAM250	0.36	0.35	0.53	0.52	0.43	0.43	0.62	0.61
PAM250SWOPT	0.38	0.37	0.57	0.56	0.44	0.44	0.65	0.64
PAM250LAOPT	0.26	0.36	0.53	0.46	0.43	0.38	0.62	0.56
GCB	0.13	0.12	0.33	0.32	0.12	0.11	0.37	0.36
GCBSWOPT	0.25	0.24	0.50	0.50	0.17	0.19	0.55	0.56
GCBLAOPT	0.14	0.15	0.39	0.31	0.16	0.093	0.44	0.38
JTT	0.34	0.34	0.53	0.51	0.47	0.46	0.61	0.60
JTTSWOPT	0.31	0.32	0.54	0.53	0.48	0.47	0.62	0.61
JTTLAOPT	0.34	0.31	0.53	0.45	0.48	0.38	0.61	0.55
